# Regarding: Staples, tension-band plates, and percutaneous epiphysiodesis screws used for leg-length discrepancy treatment: a systematic review and proportional meta-analysis

**DOI:** 10.2340/17453674.2025.43082

**Published:** 2025-02-10

**Authors:** Maria TIRTA, Søren KOLD, Ole RAHBEK

**Affiliations:** Interdisciplinary Orthopedics, Aalborg University Hospital, Denmark

*Sir,*—We thank Dr Vogt and co-authors for their engagement with our systematic review and proportional meta-analysis [1] and for acknowledging its contribution to advancing the field of leg-length discrepancy (LLD) treatment in the pediatric population.

In response to a key point raised, we wish to address a fundamental misinterpretation of our study. Specifically, regarding the categorization of the RigidTack system. We emphasize that RigidTack was treated as a distinct category, as explicitly indicated in Table 4 [1]. Therefore, the assertion that “it is methodologically incorrect to categorize the RigidTack with Blount staples” does not pertain to our study.

Additionally, we clarify that data from RigidTack implants was not included in any comparative analyses with other devices, ensuring that its unique characteristics and outcomes were preserved. As a result, the findings of the proportional meta-analysis were not influenced by the data on RigidTack.

Regarding the comment “the review’s calculation of the success rate for leg length correction using the RigidTack, as presented in Figure 4 and Tables 2 and 4, is incorrect,” we would like to clarify our approach. With regards to the success rate, we utilized the data as reported in the publication by Vogt et al. [2]: “Residual LLD ≤ 1 cm was found in 23/45 patients (51.1%), between 1 cm and 2 cm in 13/45 patients (28.9%), and > 2 cm in 9/45 patients (20.0%).” In our paper, we stated: “Two studies presented RigidTack and FlexTack staples [**47,48**], but only 1 reported a success rate, with 51% (23/45) success for RigidTack (Table 4).” Additionally, Table 4 reflects higher success rates for outcomes exceeding 2 cm correction. We acknowledge that our reporting could have been more comprehensive. However, it is important to note that RigidTack was not the primary focus of our systematic review, as it was reported in only 2 studies [2,3].

Furthermore, we acknowledge the inaccuracy in Tables 2 and 4 of our review, where it was incorrectly stated that the initial and final LLD values were not reported in their study. Upon further review, it appears this misinterpretation resulted from focusing on the figure in the publication without extracting the detailed information provided in the text. To address this, we should have included the following LLD data as reported in the Vogt et al. study [2]: “The initial leg-length discrepancy using the eight-plate was 26.7 mm and 11 mm after treatment. For the RigidTack, the initial LLD was 25.2 mm and 9.3 mm after treatment, while for Blount staples, the initial LLD was 29.3 mm and 11.4 mm after treatment.” Including this information would have provided a clearer representation of the study’s findings. However, it is important to emphasize that this clarification does not alter the conclusions of our paper.

We appreciate the authors’ constructive feedback and the valuable contributions of their research. We value this dialogue and look forward to further discussions that will enhance the understanding and treatment of leg-length discrepancies.

Finally, as there is an overlap in authorship between the cited paper (Vogt et al., 2021, Reference 47) [2] and the present letter to the editor, this is why any potential conflicts of interest have to be available and uploaded with the letter by the authors. From our side, the complete disclosure of interest forms according to ICMJE are available on the article page, doi: 10.2340/17453674.2025.43082

## References

[1] Tirta M, Hjorth M H, Jepsen J F, Kold S, Rahbek O. Staples, tension-band plates, and percutaneous epiphysiodesis screws used for leg-length discrepancy treatment: a systematic review and proportional meta-analysis. Acta Orthop 2024; 95: 415-24. doi: 10.2340/17453674.2024.41104. 39023429 PMC11257069

[2] Vogt B, Roedl R, Gosheger G, Frommer A, Laufer A, Kleine-Koenig M T, et al. Growth arrest: leg length correction through temporary epiphysiodesis with a novel rigid staple (RigidTack). Bone Joint J 2021; 103-B: 1428-34. doi: 10.1302/0301-620X.103B8.BJJ-2020-1035.R4. 34334047 PMC9948429

[3] Frommer A, Niemann M, Gosheger G, Eveslage M, Toporowski G, Laufer A, et al. Temporary proximal tibial epiphysiodesis for correction of leg length discrepancy in children: should proximal fibular epiphysiodesis be performed concomitantly? J Clin Med 2021; 10(6): 1245. doi: 10.3390/jcm10061245. 33802874 PMC8002647

